# Human Embryonic Stem Cell-Derived Mesenchymal Stroma Cells (hES-MSCs) Engraft *In Vivo* and Support Hematopoiesis without Suppressing Immune Function: Implications for Off-The Shelf ES-MSC Therapies

**DOI:** 10.1371/journal.pone.0055319

**Published:** 2013-01-29

**Authors:** Ou Li, Ariane Tormin, Berit Sundberg, Johan Hyllner, Katarina Le Blanc, Stefan Scheding

**Affiliations:** 1 Lund Stem Cell Center, Lund University, Lund, Sweden; 2 Division of Clinical Immunology Karolinska, Karolinska University Hospital Huddinge, Stockholm, Sweden; 3 Cellectis Stem Cells, Cellartis AB, Gothenburg, Sweden; 4 Division of Biotechnology/IFM, Linköping University, Linköping, Sweden; 5 Department of Hematology, Skåne University Hospital Lund, Lund, Sweden; RWTH Aachen University Medical School, Germany

## Abstract

Mesenchymal stroma cells (MSCs) have a high potential for novel cell therapy approaches in clinical transplantation. Commonly used bone marrow-derived MSCs (BM-MSCs), however, have a restricted proliferative capacity and cultures are difficult to standardize. Recently developed human embryonic stem cell-derived mesenchymal stroma cells (hES-MSCs) might represent an alternative and unlimited source of hMSCs. We therefore compared human ES-cell-derived MSCs (hES-MP002.5 cells) to normal human bone marrow-derived MSCs (BM-MSCs). hES-MP002.5 cells had lower yet reasonable CFU-F capacity compared with BM-MSC (8±3 versus 29±13 CFU-F per 100 cells). Both cell types showed similar immunophenotypic properties, i.e. cells were positive for CD105, CD73, CD166, HLA-ABC, CD44, CD146, CD90, and negative for CD45, CD34, CD14, CD31, CD117, CD19, CD 271, SSEA-4 and HLA-DR. hES-MP002.5 cells, like BM-MSCs, could be differentiated into adipocytes, osteoblasts and chondrocytes *in vitro*. Neither hES-MP002.5 cells nor BM-MSCs homed to the bone marrow of immune-deficient NSG mice following intravenous transplantation, whereas intra-femoral transplantation into NSG mice resulted in engraftment for both cell types. *In vitro* long-term culture-initiating cell assays and *in vivo* co-transplantation experiments with cord blood CD34+ hematopoietic cells demonstrated furthermore that hES-MP002.5 cells, like BM-MSCs, possess potent stroma support function. In contrast to BM-MSCs, however, hES-MP002.5 cells showed no or only little activity in mixed lymphocyte cultures and phytohemagglutinin (PHA) lymphocyte stimulation assays. In summary, ES-cell derived MSCs might be an attractive unlimited source for stroma transplantation approaches without suppressing immune function.

## Introduction

Cultured mesenchymal stromal cells (MSCs) have gained considerable interest as potential candidates for cell therapy. MSCs have a number of remarkable properties, such as high proliferation and differentiation potential, a broad cytokine production capacity and – best demonstrated for bone marrow MSCs – immune modulatory effects [1], [2]. Accordingly, MSCs have been already used clinically, for example for bone tissue repair in osteogenesis imperfecta [3], for promotion of tissue regeneration in myocardial infarction, and as immune modulators in the treatment of graft-versus host disease (GvHD) [4], [5]. Furthermore, MSCs have been demonstrated to preferentially home to tumor tissues, implicating that MSCs could be used as vehicles to effectively deliver agents with anti-tumor activity directly to the malignant cells [6], [7], [8].

MSCs are culture-derived from a small population of stromal stem cells, which are present at low frequency in adult connective tissues [9], [10]. MSCs for clinical use have thus far been derived mostly from adult bone marrow, yet there are several alternative sources such as fat tissue, cord blood and umbilical cord, amniotic membrane and other tissues [11]. Harvesting MSCs from adult tissues requires the availability of a suitable donor, and in some cases – such as bone marrow – invasive procedures need to be performed for cell harvest with potential side effects for the donors. Generally, the number of MSCs that can be generated from single-donor sources is limited, due to the restricted long-term proliferation capacity of MSCs. Furthermore, extensive culture time potentially increases the risk for inducing chromosomal aberrations and therefore, preferably low passage MSCs are used clinically [12], [13]. Moreover, cultured-derived MSCs are heterogeneous and thus difficult to standardize [14], [15].

Recently described human embryonic stem (hES) cell-derived stromal cells represent a possible alternative and unlimited source for MSCs, which might help overcome the problems with standard MSC preparations and thus enhance clinical application potential [16].

hES cells are pluripotent cells derived from the inner cell mass of the blastocyst that can be maintained in culture for an extended period of time without losing differentiation potential [17], [18]. Recently, Karlsson et al. developed an optimized protocol allowing for the simple and reproducible derivation of mesenchymal progenitor cell lines (hES-MP002.5 cells) from undifferentiated hES cells. hES-derived MP cells display the typical MSC phenotype, and – importantly – they do not form teratomas when transplanted in vivo [19], which is a major concern when ES cells are used for transplantation [18].

Previous studies mainly focused on the osteogenic capacity of hES-derived MP cells [20], [21]. Herein we report that hES-derived MP cells – in addition to standard MSC properties – possess potent hematopoietic stroma capacity in-vitro and in-vivo comparable with bone marrow-derived MSCs, however, without affecting immune function, which makes them attractive candidates for stroma transplantation approaches.

## Materials and Methods

### Ethics statement

Bone marrow donors provided their written informed consent to participate in this study. Donor bone marrow aspiration procedure and consent process were approved by the Regional Ethical Review Board in Lund, Sweden. Human cord blood cells were obtained from fullterm normal deliveries, in accordance with the protocol approved by the Regional Ethical Review Board in Lund and with written informed consent. All animal procedures were approved by the Malmö/Lund Ethical Committee on Animal Experiments, Sweden.

### Generation of bone marrow-derived MSCs

60 ml of bone marrow was aspirated from the iliac crest bone of consenting healthy donors. Bone marrow mononuclear cells (BM-MNCs) were isolated by density gradient centrifugation (LSM 1077 Lymphocyte, PAA, Pasching, Austria). BM-MNCs were seeded at 1.3×10^4^ cells/cm^2^ in non-hematopoietic (NH) expansion medium (Miltenyi Biotec, Bergisch Gladbach, Germany) and cultured at 37°C, 5% CO_2_ in tissue culture flasks (Becton Dickinson (BD), Franklin Lakes, NJ, USA). Complete medium changes were performed 3 days after initial seeding and weekly thereafter. MSCs were passaged at 70 to 90% confluency after trypsinization (0.05% Trypsin/EDTA, Invitrogen, CA, USA) and re-plated at 1,300 cells/cm^2^. Cells after seeding were designated as passage 0 MSCs, and after passaging designated as passage 1, 2, etc. Passage 3 BM-MSC were used in all experiments. MSC from a total of seven donors were used (4 males, 3 females, median age: 21 years, range 20–40).

### Culture of hES-MP002.5 cells

hES-MP002.5 cells from Cellartis (Gothenburg, Sweden) were used in this study, which were derived from female ES cells as described [19]. hES-MP002.5 cells used in this study were karyotypically normal (46, XX) as was the original hES master cell line. Cells were cultured in 0.1% Gelatin (Sigma, Stockholm, Sweden) -coated tissue culture flasks in DMEM supplemented with 10% heat-inactivated fetal bovine serum (FBS), 1% L-Glutamine, 1% Penicillin-Streptomycin, and 10 ng/ml human recombinant basic fibroblast growth factor (bFGF) (all from Invitrogen). Cells were cultured in a humidified atmosphere at 37°C and 5% CO_2_ and enzymatically passaged with TrypLETM Select (Invitrogen) every 7 days at 1∶10 split ratio. hES-MP002.5 cells used in this study were in passage 7.

### Colony-forming unit fibroblast assay

Colony-forming unit fibroblast (CFU-F) assays were performed to determine the number of mesenchymal progenitors in hES-MP002.5 cells and normal BM-MSC cultures as described before [22]. Briefly, cells were cultured in their corresponding growth medium with a complete medium change after 7 days. On day 14, the cells were washed, fixed with methanol and stained with 1% Crystal violet (Sigma). CFU-Fs were counted as colonies ≥40 cells.

### *In vitro* differentiation assays

Cultured hES-MP002.5 cells and BM-MSCs were differentiated towards the adipogenic, osteoblastic, and chondrogenic lineage as described previously [12], [13]. Briefly, cells were cultured for 14 days in AdipoDiff medium (Miltenyi) and then stained with Oil Red O (Sigma). For osteogenic differentiation, cells were cultured in osteogenesis induction medium (standard MSC medium supplemented with 0.05 mM ascorbic-acid (Wako Chemicals, Neuss, Germany), 0.1 μM dexamethasone and 10 mM β-glyerophosphate (both from Sigma)) for 21 days and calcium depositions in the cultures were detected with von Kossa staining.

Chondrogenic differentiation was induced by culturing cell pellets (2.5×10^5^ cells/pellet) for 28 days in chondrogenesis induction medium (DMEM-high glucose supplemented with 0.1 μM dexamethasone, 1 mM sodium pyruvate, 0.35 mM L-proline (all from Sigma), 0.17 mM ascorbic acid (Wako Chemicals), 1% ITS (insulin, human transferrin and selenous acid) culture supplements (BD Biosciences) and 0.01 μg/ml TGF-ß3 (R&D Systems)). Pellets were paraformaldehyde (PFA)-fixed, and frozen in Tissue-Tek OCT Compound (Sakura, Zoeterwoude, Netherlands). Cryosections were stained against aggrecan using primary goat anti-human aggrecan (R&D Systems) and TexasRed-conjugated donkey anti-goat IgG (JacksonEurope, Suffolk, UK) antibodies. Nuclei were stained with 4′,6-diamidino-2-phenylindole (DAPI, Invitrogen). Sections were analyzed with an Olympus BX51 (Olympus, Solna Sweden) fluorescence microscope and a DP70 Olympus digital camera (DP manager software Version 1.1.1.71).

### Immunophenotyping

Flow cytometry analysis of cultured BM-MSCs and hES-MP002.5 cells was performed as described previously [13], [23]. Directly conjugated antibodies used for flow cytometry (FACSCalibur, BD) included anti CD31-FITC, CD44-APC, CD45-APC, CD73-PE, CD90-APC, HLA-A, B, C-PE and HLA-DR-FITC, CD14-PE, CD19-PE, CD34-FITC, CD184-PE, CD146-PE and CD271-FITC (all from BD), and anti CD105-FITC, CD166-FITC (both from Serotec, Hamar, Norway) as well as anti SSEA4-APC (R&D Systems, Abingdon, UK). Blocking of unspecific binding was performed by incubation with human immunoglobulin, and dead cells were excluded by positive 7-Aminoactinomycin D (7-AAD, eBioscience, Inc, CA, USA) staining as described previously [24]. Data acquisition and analysis was performed using CellQuest (BD) and FlowJo software (Tree star, Ashland, OR, USA).

### Mixed lymphocyte culture (MLC) and lymphocyte mitogen stimulation assay

MLC and mitogen lymphocyte stimulation assays were performed as described previously [25], [26]. Briefly, peripheral blood mononuclear cells (PBMC) from healthy volunteers were prepared by Ficoll Hypaque separation (1,077 g/cm^3^, Axis Shield PoC AS, Oslo, Norway). For MLR, 10^5^ responder cells were co-cultured with 10^5^ irradiated (20Gy), pooled stimulator cells in complete RPMI medium for 6 days at 37°C, 5% CO_2_ in humidified air. Mitogen stimulation was performed using phytohemagglutinin (PHA, 10 ug/mL) for 4 days. Human BM-MSCs or hES-MP002.5 cells were added at various concentrations. In some experiments hES-MP002.5 cells were pre-stimulated with interferon-gamma (100 U/ml) before being added to the mixed lymphocyte culture. Cell proliferation rates were assessed by (^3^H) thymidine incorporation.

### *In vivo* migration assays

Green fluorescence protein positive (GFP+) BM-MSCs and GFP+ hES-MP002.5 cells were generated by infecting cells with GFP-encoding lentivirus VSV-G (Virus Core Facility, Lund Stem Cell Center, Lund University, Lund, Sweden) at 2∶1 MOI. 1 week after infection, GFP expressing cells were sorted by flow cytometry, followed by expansion in culture. 1×10^6^ GFP+ cultured MSCs from BM-MSCs or hES-MP002.5 cells were injected intravenously (tail vein) into irradiated (2 Gy) 8–10 week old NOD.Cg-*Prkdc^scid^ Il2rg^tm1Wjl^*/SzJ (NSG) mice (Jackson Laboratories, Bar Harbor, ME, USA). Twenty-four hours after injection, mice were sacrificed and both femurs and tibiae were harvested. Bone marrow cells were harvested by flushing all bones. Engraftment of GFP+ cells was analyzed by flow cytometry. At least 1×10^6^ events on live cells were recorded and analyzed for each sample.

### *In vivo* intrafemoral transplantation

For intrafemoral transplantations 1×10^6^ GFP+ mesenchymal stroma cells (hES-MP002.5 and BM-MSCs, respectively) were injected into irradiated (2 Gy) 8–10 week old NSG mice as described [24]. After 8 weeks, mice were sacrificed by cervical dislocation, and femurs were removed and fixed in Stefanini's fixative (2% paraformaldehyde and 0.2% picrid acid in phosphate buffer, pH 7.2) overnight at 4°C. Bone specimens were then decalcified in 0.1 M EDTA for 5 days with buffer change every day, and permealized in 20% Sucrose solution for 24 hrs. Following dehydration in increasing ethanol concentrations (70% to 100%), specimens were embedded in paraffin for analysis.

### Immunofluorescence staining of bone sections

Paraffin sections from transplanted mouse femurs were de-paraffinized and rehydrated following standard protocols [24]. Heat-induced epitope retrieval was applied using citrate buffer, pH 6 (Target Retrieval Solution, Dako, Glostrup Denmark) for 30 min at 98°C. Sections were blocked/permeabilized with DPBS 0.3% Triton X-100 (Sigma), 10% normal goat serum, 0.1% sodium azide and 0.1% cold fish skin gelatin (Sigma) and stained for 1 hour RT or overnight at 4°C with anti-GFP (Abcam, Cambridge, UK) and anti human CD31 (clone SP38, Spring Bioscience, CA, USA) antibodies. After washing, secondary staining was performed (1 h, room temperature) with goat anti-chicken IgG-Alexa Flour 488 and goat anti-mouse IgG1-Alexa Flour 555 (both Invitrogen). TO-PRO-3 (Invitrogen) was used as nuclear stain. Pictures were taken on a DMRE confocal microscope (Leica, Mannheim, Germany) equipped with green helium/neon, standard helium/neon and argon lasers, using Leica Confocal Software v2.61.

### Long-term culture initiating cell (LTC-IC) assays

In-vitro stroma supporting capacities of BM-MSCs and hES-MP002.5 cells were assessed with standard LTC-IC assays [24]. 3×10^5^ MSCs were plated in collagen-coated 35-mm culture dishes and irradiated the following day (16 Gy). Primary human cord blood CD34+ cells were isolated from donor cord blood by magnetic activated cell sorting (MACS) on a MiniMACS separator with MS columns following incubation with CD34 MicroBeads according to the manufacturer's instructions (Miltenyi). Forty eight hours after irradiation, 3000 selected CD34+ cells were seeded and cultured in MyeloCult H5100 media (Stem Cell Technology) containing 1×10^−3^ mM Hydrocortisone (Sigma) with weekly half-medium changes. After 6 weeks, cells were harvested by trypsinization and assayed for hematopoietic colony formation. Briefly, cells were resuspended in IMDM +2% FBS (both Invitrogen) and seeded into methylcellulose medium (HSC-CFU complete with Epo, Miltenyi). After two weeks culture hematopoietic colonies were analyzed under a microscope according to standard criteria.

### *In vivo* stroma support (co-transplantation) assays

1×10^6^ hES-MP002.5 cells and BM-MSCs, respectively, were injected intrafemorally (i.f.) into the left femur of irradiated (2 Gy) 8–10 week old NSG mice. Immediately following the i.f. injections, mice received an intravenous injection of 5×10^5^ MACS-enriched human cord blood CD34+ cells. Control mice were i.f. injected with medium. Six weeks later, mice were sacrified and left (MSC injected) and right femurs (MSC non-injected) were harvested and analyzed separately. Bone marrow cells were extracted by crushing the bone and repetitive rinsing with PBS. Engraftment of human hematopoietic cells was analyzed flow cytometrically utilizing human specific CD45-APC, CD34-FITC, CD14-PerCP and CD19-PE antibodies and corresponding mouse IgG1-FITC and IgG1-PE isotype controls (all from BD). Engraftment rates between MSC and non-MSC transplanted femurs of mice in the same treatment group and different treatment groups were compared using the paired T-test and ANOVA with post hoc Tukey's test, respectively (GraphPad Prism, GraphPad Software, La Jolla, CA, USA).

## Results

### hES-MP002.5 generated CFU-F in-vitro

**Figure 1:**
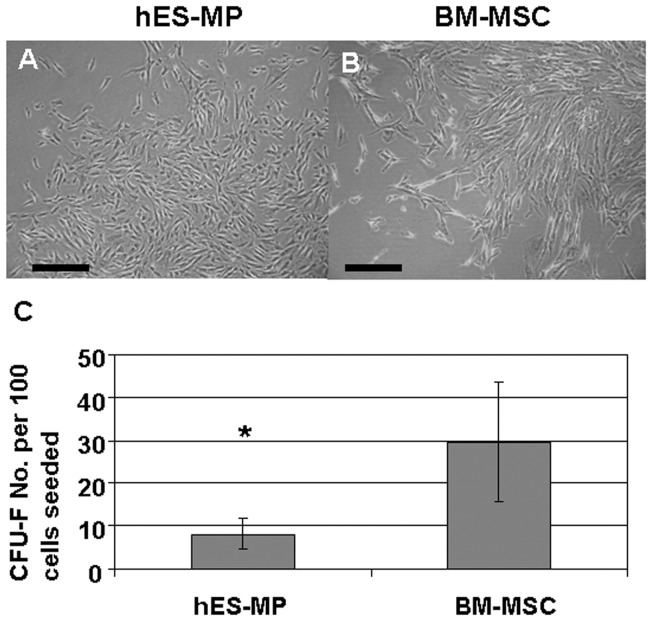
Cell morphology and CFU-F frequency of cultured hES-MP002.5 cells compared with BM-MSCs. hES-MP002.5 cells and BM-MSCs, respectively, were assayed for CFU-F. (A–B) Except for differences in cell size, colonies derived from hES-MP002.5 cells (A) showed a similar morphology compared to CFU-F assayed from standard bone marrow-derived MSCs (B). Scale bars indicate 100 μm. (C) Frequencies of CFU-F in standard BM-MSCs preparations are higher when compared with hES-MP002.5 cells. Results are shown as mean number of CFU-F numbers per 100 seeded cells ± standard deviation. Data were analyzed by student's *t test*. n = 6. * p<0.05.

Single cell suspensions were prepared from hES-MP002.5 cells and BM-MSCs and tested for their mesenchymal progenitor growth using the standard CFU-F assay. Colonies derived from hES-MP002.5 cells were morphologically similar to normal BM-MSCs derived CFU-F colonies (Fig. 1A, B). The colony-forming capacity of hES-MP002.5 cells was enumerated as 8±3 CFU-F per 100 cells whereas BM-MSCs gave rise to 29±13 CFU-F per 100 cells (Fig. 1C).

### hES-MP002.5 cells showed a similar immunophenotye as BM-MSCs

**Figure 2:**
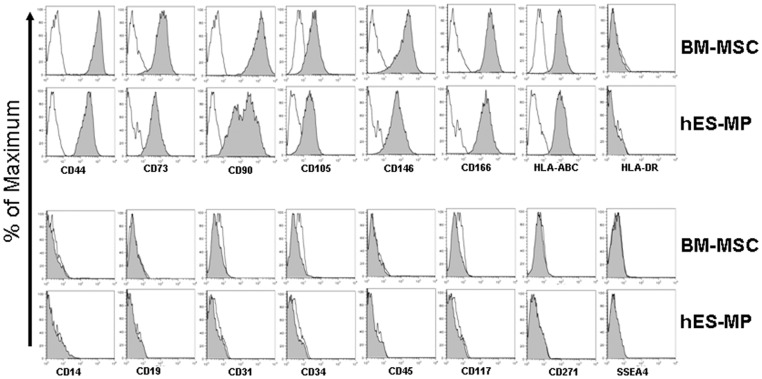
Surface marker expression profiles of hES-MP002.5 cells and BM-MSCs. hES-MP002.5 cells and BM-MSCs were trypsinized and stained with different combinations of antibodies for flow cytometric analysis. The data are presented as histograms. The percentage of the maximum of the number of cells in each channel is shown on the y-axis. Samples are presented in the shaded plots, corresponding isotype controls are shown as open lines. hES-MP002.5 cells and BM-MSCs showed comparable surface marker expression profiles, except for differences in CD90 expression. Representative histograms of one of three independent experiments are shown.

Multi-color FACS analysis was performed to assess co-expression of signature mesenchymal and stromal cell surface markers on BM-MSCs and hES-MP002.5 cells. As illustrated in Figure 2, both cell types were positive for CD44, CD73, CD90, CD105, CD146, CD166 and HLA-ABC, and stained negative with antibodies against HLA-DR, CD14, CD19, CD31, CD34, CD45, CD117, CD271, and SSEA-4. The only obvious difference in surface marker expression was observed for CD90. Here, hES-MP002.5 cells showed a bimodal distribution of CD90 expressing cells whereas the distribution of BM-MSCs was clearly unimodal (Fig. 2).

### Differentiation potential of hES-MP002.5 cells *in vitro*

**Figure 3:**
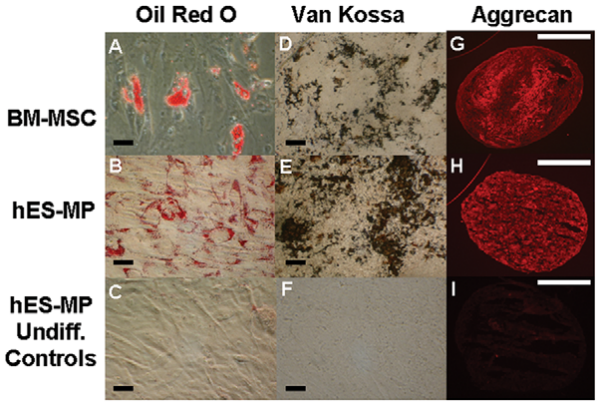
Differentiation capacity of hES-MP002.5 cells and BM-MSCs. Cultured cells were differentiated towards the adipogenic (left column), osteogenic (middle column) and chrondrogenic lineage (right column). Formation of adipocytes was confirmed by Oil-Red-O staining of lipid vacuoles in differentiated BM-MSCs (A) and in hES-MP002.5 cells (B). Undifferentiated hES-MP002.5 cells controls are shown in (C). Osteoblastic differentiation was demonstrated by calcium deposits stained by von Kossa staining of differentiated BM-MSCs (D) and hES-MP002.5 cells (E), but not in the undifferentiated hES-MP002.5 cells control (F). Differentiation into chondrocytes was confirmed by staining for aggrecan (red) in sectioned chondrocyte pellets derived from differentiated BM-MSCs (G) and hES-MP002.5 cells (H). Undifferentiated hES-MP002.5 cells are shown as control in (I). Black scale bars indicate 50 μm; white scale bars indicate 500 μm.

By definition, mesenchymal stromal cells are capable of multilineage differentiation, typically into adipocytes, chondrocytes and osteoblasts. To test whether hES-MP002.5 cells possess the same differentiation potential as BM-MSCs, cells were exposed to appropriate induction media for adipocyte, osteoblast and chondrocyte differentiation, respectively. Successful adipocytic differentiation of control BM-MSCs was verified by the staining of lipid-containing vacuoles with Oil-Red-O (Fig. 3 A). In comparison, adipocytic differentiation induction of hES-MP002.5 cells yielded a higher number of smaller aggregates of lipid droplets (Fig. 3, B). Osteoblastic differentiation potential was demonstrated by staining of calcium deposits using von Kossa staining (Fig. 3, D–F). Comparing with BM-MSCs, hES-MP002.5 cells showed higher osteoblastic differentiation potential as demonstrated by stronger von Kossa staining. Differentiation towards the chondrogenic lineage of both, BM-MSCs and hES-MP002.5 cells was demonstrated by immune-staining for the proteoglycan aggrecan (Fig. 3, G–I).

### hES-MP002.5 cells are considerably less potent immune-modulators than BM-MSCs

**Figure 4:**
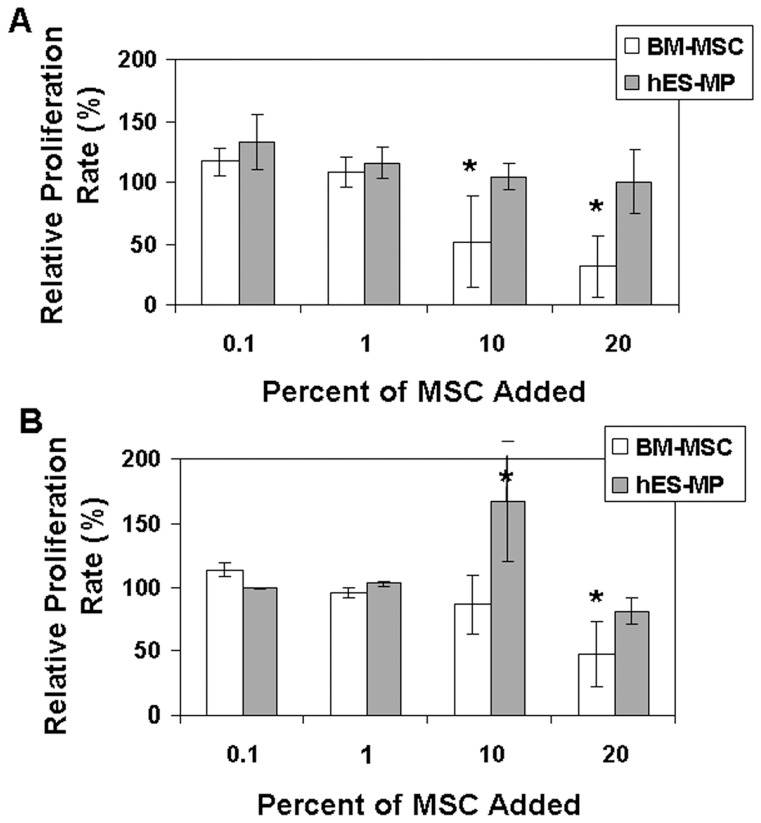
Immune-modulatory functions of BM-MSCs and hES-MP002.5 cells. BM-MSCs or hES-MP002.5 cells were added to mixed lymphocyte cultures (A) and PHA-stimulated lymphocyte cultures (B) [22] to examine their effect on allogeneic- and mitogen-induced lymphocyte proliferation, respectively. The percentage of stromal cells added to the cultures ranged from 0% (control) to 20% (x-axis). Lymphocyte proliferation rates are expressed as percentages of controls, i.e. cultures to which no MSCs were added. BM-MSCs for these experiments were generated as described previously [26]. Data are shown as mean ± SD of at least 3 independent experiments. Statistical differences compared to controls are indicated as *: p<0.05 (student *t test*).

To investigate whether the two stromal cell preparations have similar *in vitro* immune suppressive properties, hES-MP002.5 cells and BM-MSCs were added to mixed lymphocyte cultures and PHA-stimulated lymphocyte assays. Unlike BM-MSCs, hES-MP002.5 cells showed no inhibitory effect on proliferation rates in allogen-stimulated mixed lymphocyte cultures, whereas BM-MSCs as expected clearly inhibited proliferation (Fig. 4 A). BM-MSCs were also clearly suppressive at higher cell doses in mitogen-stimulated cultures (Fig. 4 B). Here, a slight inhibition of proliferation was observed at the highest hES-MP002.5 cells cell dose, i.e. 20%. However, lower doses of hES-MP002.5 cells did either not affect or even stimulate lymphocyte proliferation (Fig. 4 B). Interferon-γ pre-treatment has been reported to enhance/trigger the immune-suppressive effect of MSCs [25]; however, this was not the case when hES-MP002.5 cells were exposed to interferon-γ (data not shown).

### Neither BM-MSCs nor hES-MP002.5 cells home to the bone marrow of NSG mice after intravenous injection

In order to test whether the different MSC preparations are capable of *in vivo* homing to the bone marrow following intravenous injection, NSG mice were injected intravenously with GFP-expressing hES-MP002.5 cells or GFP-marked BM-MSCs. Flow cytometric analysis of bone marrow cells harvested 24 hour after injection revealed that GFP+ cells were not detectable, regardless of whether hES-MP002.5 cells or BM-MSCs were used. On the other hand, human cells were clearly detectable in control animals receiving intrafemoral injections (data not shown).

### hES-MP002.5 cells as well as BM-MSCs are capable of in-vivo bone marrow engraftment in NSG mice when injected intrafemorally

MSCs were recently reported to play an important role in the bone marrow hematopoietic micro-environment [24], [27], [28]. Therefore, the ability to engraft long term in the bone marrow is the key for clinical approaches aiming to provide effective stromal support.

**Figure 5:**
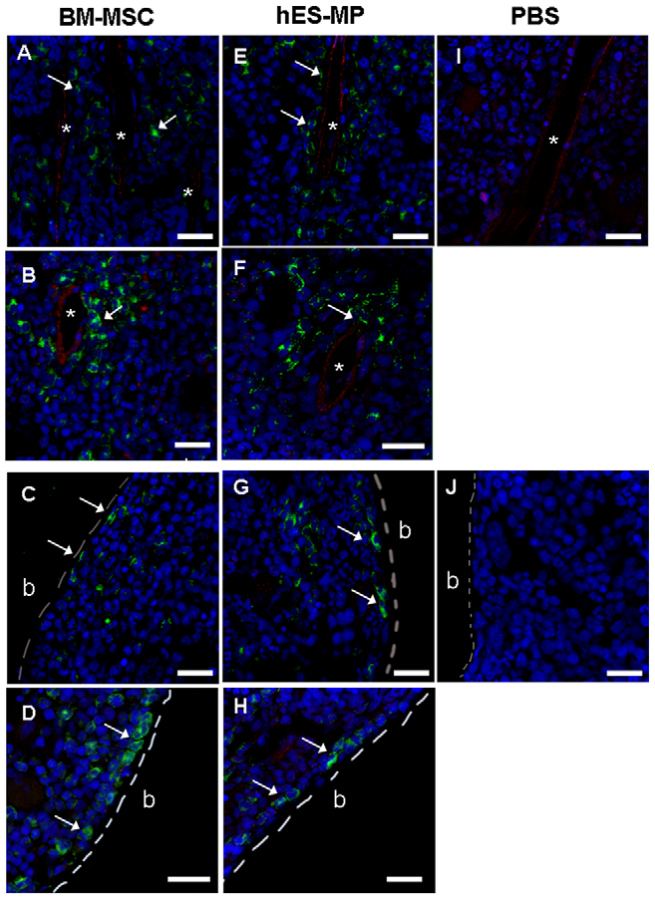
Intrafemoral transplantation of MSCs into immunodeficient NSG mice. GFP+ MSCs generated from cultured BM-MSCs (left panel) and hES-MP002.5 cells (middle panel) cells, respectively, were transplanted intrafemorally into NSG mice. PBS (right panel) was used as transplantation controls. Eight weeks later, the analysis of femur sections was performed by immunostaining followed by confocal microscopy. GFP+ BM-MSCs as well as GFP+ hES-MP002.5 cells (green, marked by white arrows) were detected in perivascular regions surrounding the endothelium (CD31, red) (A, B, E, F), and as bone-lining cells near the endosteal surface, respectively (C, D, G, H). Nuclei were stained with TO-PRO3 (blue). Scale bar  = 25 μm. * blood vessel; **b** bone.

Intra-femoral transplantation into NSG mice using GFP expressing hES-MP002.5 cells and BM-MSCs revealed that GFP+ cells were clearly detected in femur bone sections 8 weeks after transplantation, as indicated by immune-staining with anti-GFP antibody. GFP+ cells in both, hES-MP002.5 cells as well as BM-MSCs transplanted bone marrows showed similar morphologies (Fig. 5). Furthermore, localization of transplanted cells was comparable, i.e. engrafted cells mainly clustered around blood vessels (counter-stained by anti-CD31 antibody, Fig. 5 A, B, E, F) and near to the endosteal surface of the femurs (Fig. 5 C, D, G, H).

### hES-MP002.5 cells, like BM-MSCs possess potent hematopoietic stroma function *in vitro* and *in vivo*

**Figure 6:**
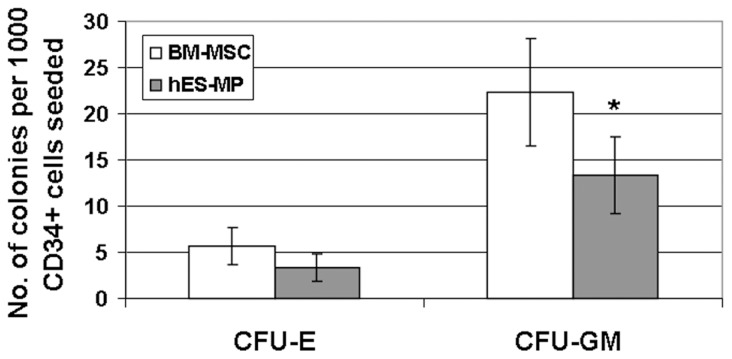
In-vitro stromal function of BM-MSCs and hES-MP002.5 cells in LTC-IC assays. Cord blood derived CD34+ cells were co-cultured on irradiated stromal feeder layers, i.e. either on BM-MSCs or hES-MP002.5 cells. After 6 weeks, cells were harvested and analyzed for colony formation in standard methylcellulose. Numbers of CFU-E and CFU-GM generated per one thousand seeded CD34+ cells are shown as mean ± standard deviation from three independent experiments (n = 3). * *p*<0.05.

To test for stroma function *in vitro*, standard LTC-IC assays were set up using irradiated hES-MP002.5 cells or BM-MSCs as feeder cells. After 6 weeks of co-culture, hematopoietic colonies were generated from MACS-enriched cord blood CD34+ cells on both feeder cell layers, with BM-MSCs showing a more potent in-vitro stroma function than hES-MP002.5 cells (Fig. 6).

**Figure 7:**
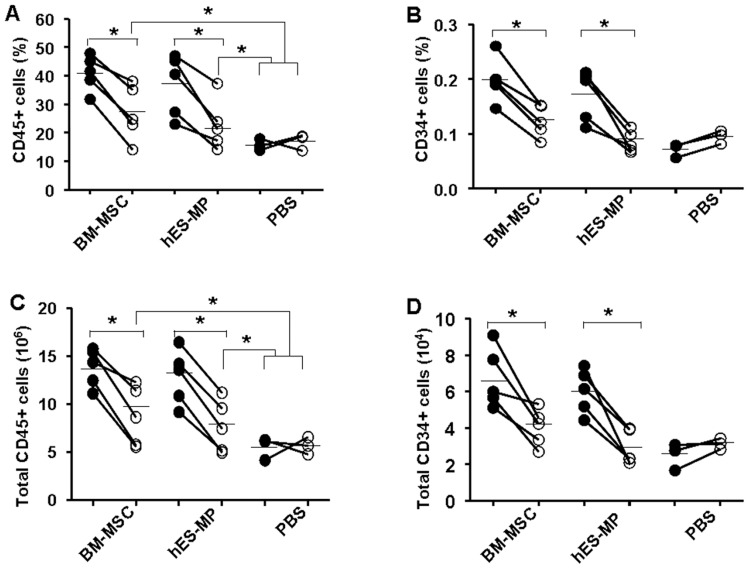
Analysis of in-vivo stromal function of BM-MSCs and hES-MP002.5 cells by co-transplantation with CD34+ hematopoietic cells in NSG mice. NSG mice received intrafemoral transplantation of stromal cells into the left femur followed by intravenous injections of cord blood derived CD34+ cells. After 8 weeks, cells from both femurs were harvested separately and stained with anti human CD45 (A, C) and CD34 (B, D) antibody and analyzed by flow cytometry. Control mice received intrafemoral PBS only. Data are presented as percentages (A, B) as well as absolute numbers (C, D) of human cells in the femurs of individual mice. Lines connect data from left (injected, BM-MSC+, hES-MP002.5 cells+, PBS+, shown with solid dots) and right (non-injected, BM-MSC-, hES-MP002.5 cells-, PBS-, shown with circles) femurs of the same animal. Mean values were indicated as horizontal lines. Statistical differences are indicated as: * *p*<0.05.

To test for stroma function *in vivo*, hES-MP002.5 cells or BM-MSCs were transplanted into the left femur of irradiated NSG mice followed by co-transplantation of MACS-enriched human cord blood CD34+ hematopoietic cells. After 6 weeks, percentages (Fig. 7A, B) as well as total numbers (Fig. 7 C, D) of human CD34+ and CD45+ cells were higher in the hES-MP002.5 cell-transplanted, left femur compared with the non-transplanted femur (right) in the same mouse. Similar differences were observed when BM-MSCs were used, indicating that the in vivo stroma capacities of both cell types were comparable (Fig. 7). Furthermore, no differences were observed in the fractions of CD45/CD14 and CD45/CD19 positive cells between the BM-MSC and hES-MP002.5 groups showing that the production of myeloid cells and B-cells was not skewed by the different stromal cell types. Notably, higher numbers of CD45+ cells but not CD34+ cells were detected in the non-transplanted femur in MSC transplanted mice compared with intrafemoral PBS injection controls (Fig. 7), a difference that is likely to reflect the paracrine activity of the orthotopically transplanted stromal cells.

## Discussion

Cultured mesenchymal stroma cells (MSCs) have a high potential for novel cell therapy approaches in clinical transplantation due to their intriguing properties, i.e. high proliferation and multi-lineage differentiation capacity, stroma support function and immune-modulation [4], [29], [30], [31], [32], [33].

Currently, bone marrow cells are the major source for MSC therapies. However, bone marrow MSCs – as other MSC preparations derived from single donor adult stroma stem cell sources – are primary cells, which have certain properties that unfortunately limit a broader clinical application in larger cohorts of patients. First, the availability of primary bone marrow cells is dependent on (healthy volunteer) donors, who are willing to undergo a relatively harmless but nevertheless invasive bone marrow harvest procedure. Secondly, and importantly, the number of BM-MSCs that can be generated from a single donor is limited and usually does not allow treating more than one or two patients. Furthermore, growth characteristics and cell yields of BM-MSC preparations vary among individual preparations and MSC proliferation is donor age dependent [34]. In addition, we and others have reported biological differences even within BM-MSCs culture-derived from a single donor [35], indicating that standardization of a primary adult stem cell-derived MSC products is difficult. Last, BM-MSCs can only be passaged for a limited number of times, after which they show a marked reduction in proliferation and differentiation potential. In addition, genetic instability and the risk of developing chromosomal aberrations increase with prolonged time in culture [36].

Therefore, an alternative and unlimited source of MSC with high potential for standardization for cell-based therapies is highly desirable. Recently developed hES cell-derived MSCs may represent such a potential alternative MSC source. These cells, named hES-MP002.5 cells, were derived by direct differentiation of hES cells in a feeder-free culture system using DMEM medium containing 10% FBS and 10 ng/ml basic fibroblast growth factor (bFGF). Under these conditions, growth of a stromal cell population with typical MSC properties was observed after two to three passages [19].

We report herein a detailed biological study of these cells, demonstrating that hES cell-derived MSCs have a similar morphology and a typical MSC surface marker expression pattern compared to standard human bone marrow-derived MSCs [37], which is in accordance with recently reported results [21]. The only difference between the two cell types was the bimodal expression of CD90 in hES-MP002.5 cells, which has also been reported for human placenta-derived MSCs [38]. Interestingly, CD90 expression has been demonstrated to decrease with osteoblastic differentiation [39] and thus, CD90 expression differences may reflect differences in differentiation potential towards the osteoblastic lineage within hES-MP002.5 cells. We have not formally addressed possible heretogeneity of ES-MSC in the current work and, thus, these important considerations remain to be investigated.

hES-MP002.5 cells in our experiments showed a lower CFU-F potential compared with BM-MSCs, which might be due to differences in passage number. However, considering the various CFU-F numbers reported previously for BM-MSCs [23], [40], hES-MP002.5 cells still showed reasonable CFU-F generation capability even in higher passages.

The *in vitro* hES-MP002.5 cells differentiation potential towards chondrogenesis was comparable with that of BM-MSCs, whereas on the other hand hES-MP002.5 cells showed higher osteogenic potential and a different pattern of adipocytic cells. These results are in line with previous reports with this specific hES-MP002.5 cells cell line as well as with the results obtained with other hES-derived MSCs [20], [21]. It was furthermore shown that the superior osteogenic potential of hES-MP002.5 cells is due to signaling pathway differences [20], however, the reason for the differences in adipogenic differentiation has not yet been identified and needs to be investigated further.

Recent studies indicate that MSCs are key constituents of the bone marrow microenvironment, thus playing an important role for the regulation of hematopoietic stem cell (HSC) self-renewal and differentiation [24], [27], [28]. Because of their known stroma supporting capacity, BM-MSCs have been suggested to be able to enhance HSC engraftment after transplantation [33]. Accordingly, studies in humans as well as xenotransplantation experiments showed that co-transplantation of human MSCs and HSCs resulted in increased chimerism and accelerated hematopoietic recovery [41], [42], [43]. Interestingly, bone marrow homing of *in vitro* cultured MSCs have – with the exception of *ex vivo* homing receptor-engineered MSCs [44] – not yet been demonstrated conclusively. Thus, effects on hematopoiesis observed after i.v. administration are likely to be due to the paracrine factors released by cultured MSCs.

Accordingly, our results demonstrate that neither hES-MP002.5 cells nor BM-MSCs homed to the bone marrow of NSG mice after intravenous injection, at least not to such an extent that homing would have been detectable by flow cytometry analysis of stably-transfected GFP+ MSCs.

On the other hand, when MSCs were injected intra-femorally, both hES-MP002.5 cells and BM-MSCs engrafted, and localization of the transplanted cells was comparable with the distribution of primary BM-MSCs in-situ, i.e. cells were primarily found perivascularly and also endosteally [24], [45].

The hematopoietic stroma capacity of the two cell populations was tested *in-vitro* with the LTC-IC assay and *in-vivo* by co-transplantation of human CD34 hematopoietic cells (i.v.) and stroma cells (i.f.) into immunodeficient NSG mice. The results of both assays clearly demonstrated that hES-MP002.5 cells and BM-MSCs have potent stroma supporting capacity. It is worth mentioning that the injection of stromal cells in one femur also enhanced hematopoiesis in the non-stroma-injected femur when compared to control mice without stroma cell support. This observation strengthens the assumption that transplanted stroma cells do not only act locally by direct interaction with hematopoietic cells but also act in a paracrine fashion to enhance hematopoiesis even in a distant, non-stroma-injected site. An alternative explanation would be that intrafemorally injected stromal cells migrated and homed to the opposite femur. However, this appears to be rather unlikely given the negative results of our migration experiments. The potent stroma-supportive activity of hES-MP002.5 cells is an important property of this novel type of mesenchymal ES-cell derived cells. However, whether or not all ES-cell derived mesenchymal cell lines share this property has not yet been reported and, certainly, this is a relevant topic for future investigations [19].

One of the most prominent current clinical applications of BM-MSCs is their use as immune-modulators in diseases such as Graft versus Host Disease (GvHD), inflammatory bowel disease, and others [46]. MSCs exert their immune effects by direct cell-cell contact as well as by other mechanisms, such as production of transforming growth factor β-1 (TGF-β1), indoleamine 2,3-dioxygenase (IDO), prostaglandin E2 (PGE2), nitric oxide (NO), and other substance as well as by recruiting other immune-suppressing networks [46].

Our results demonstrate that hES-cell derived stromal cells – at least in vitro – have only little or no effect on allo-antigen as well as mitogen-stimulated lymphocyte proliferation (Fig. 4). Even when pretreated with interferon-γ, which was previously shown to increase HLA-DR expression and to enhance immune-suppressive activity of fetal MSCs [47], [48], hES-MP002.5 cells remained non-immunosuppressive. Accordingly, others have shown that HLA-DR expression of hES-MP002.5 cells does not increase upon interferon-γ treatment [21]. Thus, in contrast to BM-MSCs, hES-MP002.5 cells are most likely not suitable for cell therapy approaches that aim to modulate immune function.

However, there are other clinical applications which do not depend on the immune-suppressive effects of MSCs and for some applications, this “side effect” might even be harmful. For example, there is no need for immune-modulation when administering MSCs to accelerate hematopoietic recovery after autologous transplantation. Furthermore, patients undergoing allogeneic transplantation for high-risk leukemia would certainly profit from the stroma-supporting effects of additionally transplanted MSCs leading to a faster hematopoietic recovery [41], [42], [43]. However, additional MSC-induced immune suppression in the early post-transplant phase might dampen NK graft-versus tumor activity, thus increasing the risk for relapse [49]. In addition, based on their ability to migrate to and invade tumor tissues, MSCs have also been proposed as cellular vector systems in anti-tumor therapy approaches, e.g. for the delivery of oncolytic viruses or other substances with anti-cancer activity [6], [7]. For theses applications the use of immuno-suppressive MSC preparations would post a safety concern as this may affect the host anti-tumor immune response possibly resulting in enhanced tumor growth and metastasis [50]. Non-immunosuppressive MSC products such as the hES-MP002.5 cells described herein, would here certainly be the preferable choice of cells over MSC preparations with immune-suppressive potential. However, whether or not non-immunosuppressive hES-derived MSC can be transplanted across HLA barriers in a similar way as bone marrow-derived cells is not clear at the moment. Our experiments showed that both cell types lack expression of class II HLA molecules, however, transplantibility of hES-MP cells remains to be investigated in appropriately designed studies.

In summary, our study shows that the recently developed hES cell-derived MSC cell line hES-MP002.5 cells possess similar biological and functional properties compared to conventional BM-MSCs, except for the immune-modulatory effects. Based on the fact that hES-MP002.5 cells can be reliably and safely produced from established ES cell lines, hES-MP002.5 cells are an attractive unlimited source for stroma transplantation approaches in clinical situations when immune suppression is either not required or even potentially dangerous.
